# A Rare Case of Purulent Pericardial TB

**DOI:** 10.7759/cureus.5356

**Published:** 2019-08-09

**Authors:** Muhammad Izzad Johari, Ahmad Wazi Ramli, Firdaus Mat Lawi, Mohamad Ariff Hafizi Bin Fouzi, Kurnia Prima Sawai Suardi

**Affiliations:** 1 Cardiology, Hospital Sultanah Nur Zahirah, Terengganu, MYS; 2 Internal Medicine, Hospital Sultanah Nur Zahirah, Terengganu, MYS; 3 Cardiology, Universiti Sultan Zainal Abidin, Terengganu, MYS

**Keywords:** tuberculosis, pericardial disease, steroids, constrictive pericarditis

## Abstract

Pericardial effusion is a common disease and tuberculosis (TB) accounts up to 4% of acute pericarditis with up to 7% of tamponade case. Accurate diagnosis is important while quick intervention can be life-saving. A case was reported involving a 73-year-old man who presented with reduced effort tolerance for one-month duration. During hospitalization, further workup revealed the presence of massive purulent pericardial effusion with evidence of tamponade. TB gene expert was positive in aspirated pericardial fluid and the patient was treated promptly using a combination of anti-TB drugs with the addition of steroid therapy.

## Introduction

Tuberculosis (TB) infection is an important airborne infections disease leading to an epidemic especially in developed countries and one of the top 10 causes of death worldwide. In 2017, 10 million people fell ill with TB, and 1.6 million died from the disease with 0.3 million were HIV patients. The largest number of new TB cases occurred in the South-East Asia and Western Pacific regions, with 62% of new cases, followed by the African region, with 25% of new cases. About 1-2% of pulmonary tuberculosis patients were to have tuberculous pericarditis [1]. In developed countries, TB pericarditis accounts around 4% of acute pericarditis. In one case series in Spain, 294 immunocompetent patients presented with acute pericarditis, in whom the cause was not apparent at initial presentation, 4% (13 patients) of cases were tuberculous pericarditis, five of 13 patients were having cardiac tamponade and constrictive pericarditis developed in six patients [2]. Quick treatment of tuberculous pericarditis can be lifesaving and it requires a fast and accurate diagnosis for disease, but it frequently can be difficult [3]. TB purulent pericarditis (pyopericardium) is unusual presentation of TB and has been reported in 6.98% of the cases [4]. Pyopericardium has been documented in <3% of the cases of large TB pericardial effusions, even in the high-prevalence areas of TB and human immunodeficiency virus infection [5].

To the best of the researchers’ knowledge, there are no available data in Malaysia on the prevalence of tuberculous pericarditis. We herein report the case of disseminated tuberculosis with pericardial empyema. This case hopes to illustrate the significance to identify and recognize tuberculous pericarditis in a TB endemic region such as Malaysia and early detection of atypical presentation of TB.

## Case presentation

A 73-year-old man with background history of bilateral eye cataract presented with fever for one week and associated with chills and rigor. Along with the fever, he had cough with whitish sputum, lethargic with reduced oral intake and intermittent right upper quadrant abdominal pain. The pain was described as dull aching, worsen with lying down position however not associated with acid brash, nausea, vomiting and yellowish discoloration of the skin and mucous membrane. Further history revealed that the patient had chronic heart failure symptoms and reduced effort tolerance for months, giving NYHA class 2. Other than that, there was no complaint of constitutional symptoms and contact with pulmonary TB. The patient was treated for acute cholecystitis and did cholecystectomy. Blood culture and tissue culture from the gallbladder showed no growth. In regards of his reduced effort tolerance, transthoracic echocardiography was done, and the result revealed global pericardial effusion with no sign and symptom of cardiac tamponade; hence no pericardial tapping was done at that time.

One-month post cholecystectomy, the patient complained of worsening symptoms of lethargy, chest pain, shortness of breath on exertion, associated with orthopnea. Echocardiography showed massive pericardial effusion with RV collapse on diastole. Emergency pericardiocentesis was performed. Pericardial collection appeared to be yellowish and turbid. Pericardial fluid was sent for microbiological examination. The Ziehl-Neelsen (ZN) stained smears showed no evidence of acid-fast bacilli (AFB). Culture on Lowenstein Jensen (LJ) media showed no evidence of colonies suggestive of Mycobacterium tuberculosis. TB genome detection from pericardial fluid showed to be positive for TB. Three early morning sputum samples were examined by ZN stain and LJ culture but were negative for AFB and the culture showed no growth after eight weeks of incubation.

The patient was tachypnoeic, toxic looking and in sepsis. His vital signs showed the following: respiratory rate of 24 breaths/min, tachycardia of 110 beats/min, blood pressure of 127/68 mmHg and temperature of 38°C. Clinical examination supported the diagnosis of cardiac tamponade. Jugular venous pulse was raised and on auscultation, the heart sounds were muffled and associated with a pericardial rub. There was no peripheral oedema, cyanosis, pallor, icterus or hepatosplenomegaly.

**Figure 1:**
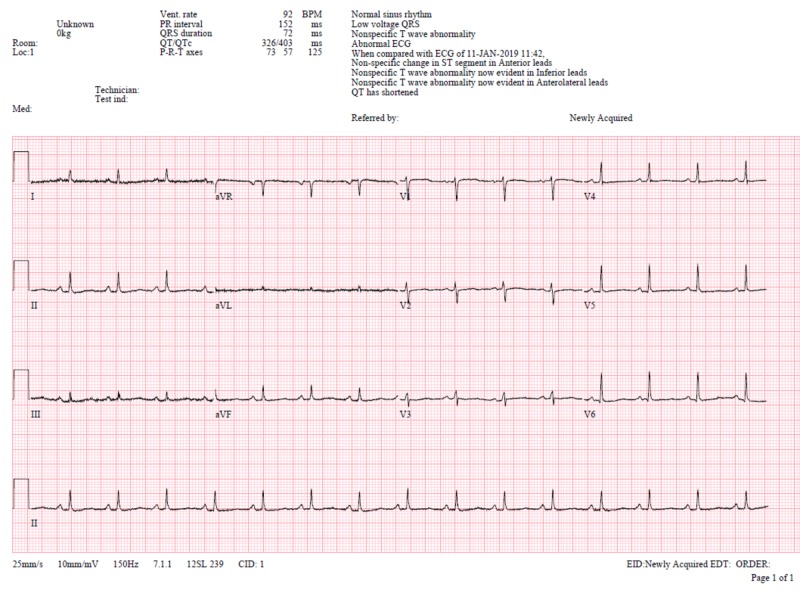
ECG upon admission showing low voltage QRS complexes. ECG: Electrocardiography

**Figure 2:**
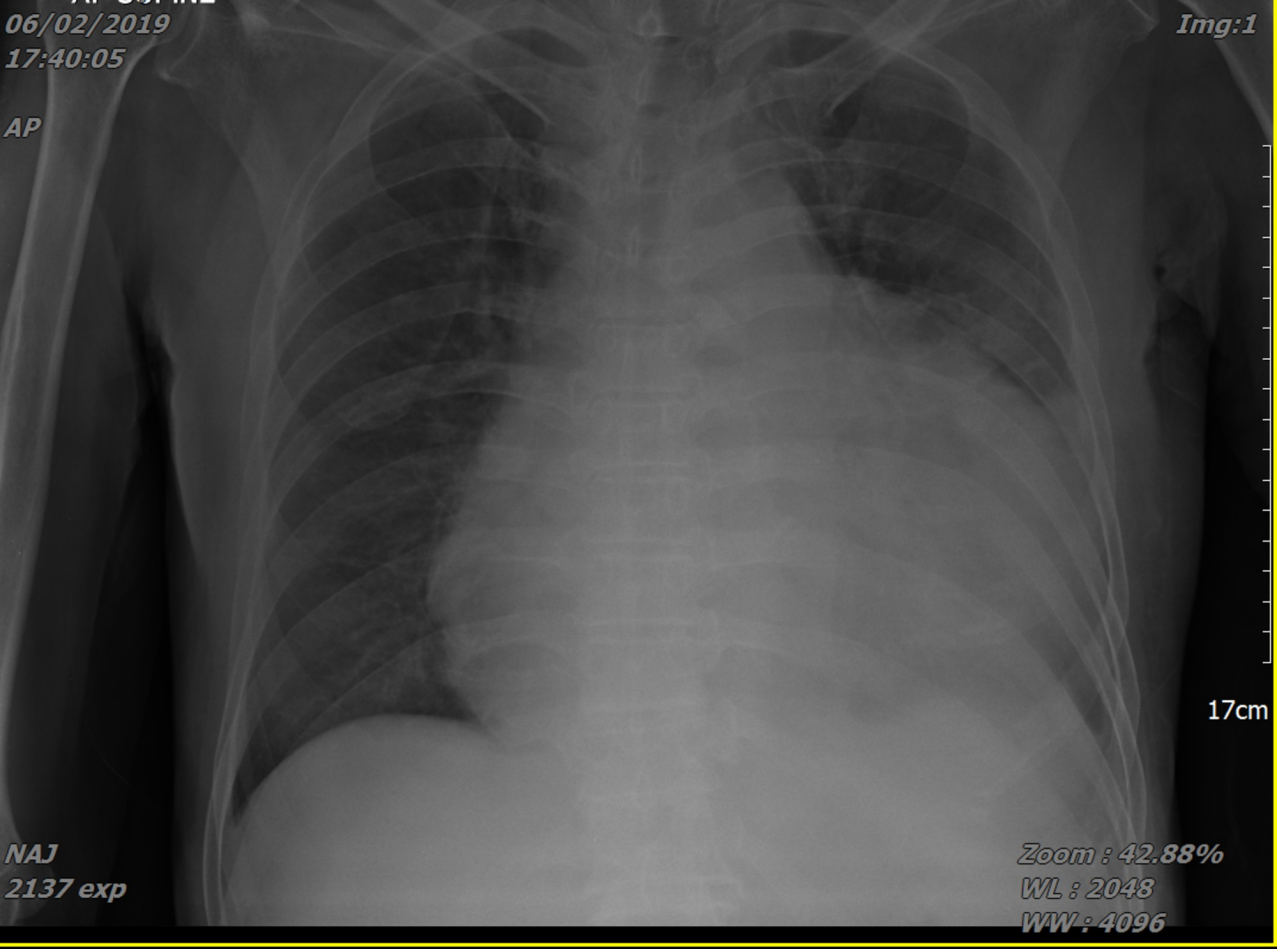
Chest radiograph upon admission showing cardiomegaly, pulmonary congestion and bilateral pleural effusion.

**Figure 3:**
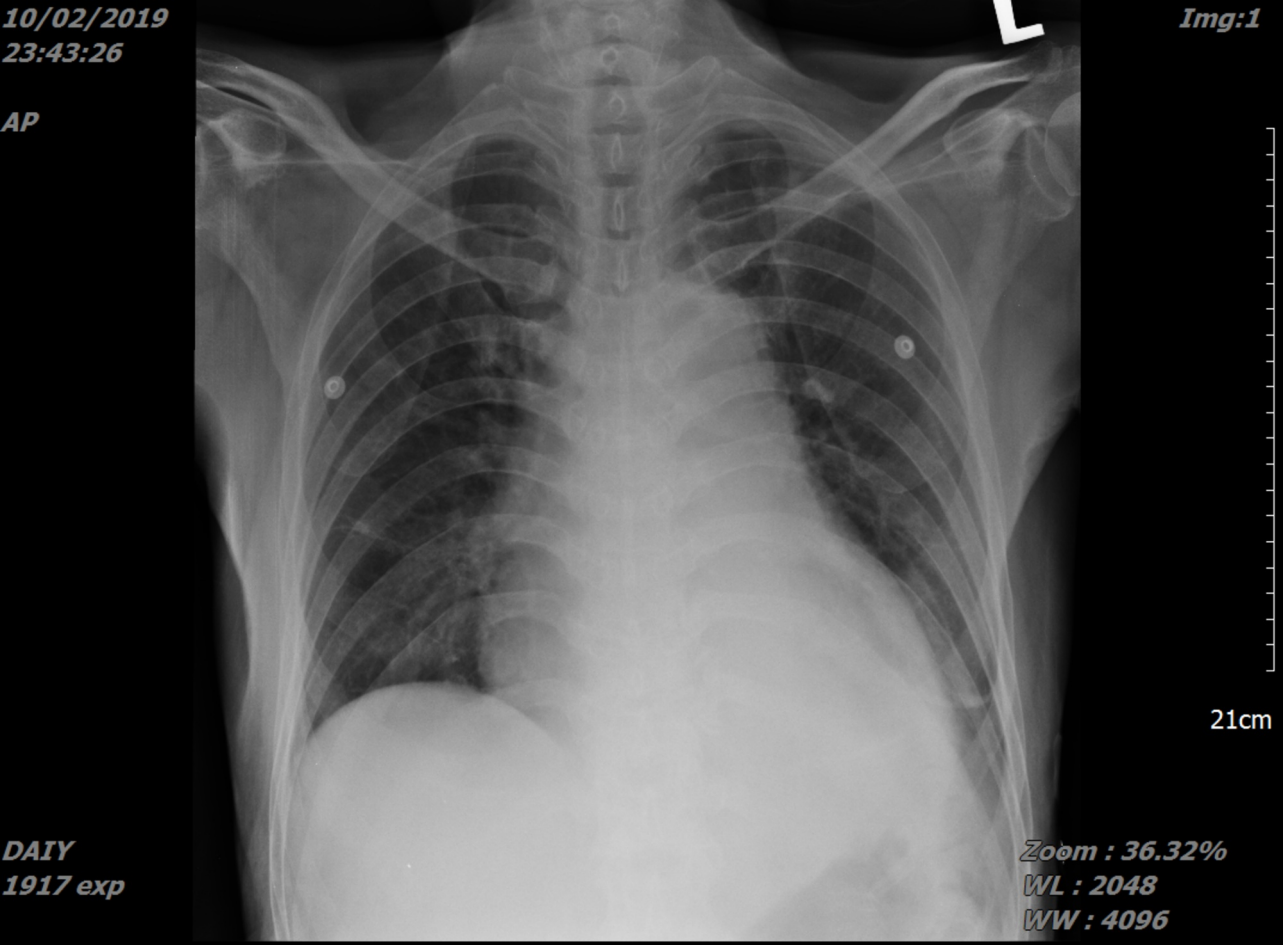
Chest radiograph after emergency pericardiocentesis.

Laboratory test revealed haemoglobin of 11 g/dl, white blood count of 7.8 x 10^9^/L with 80% neutrophils and 9.5% of lymphocytes. C-reactive protein was high, 144.5 mg/L. The patient was seronegative for HIV. Tuberculin skin test (TST) was positive (16 mm). Electrocardiography (ECG) showed low voltage complex (Figure 1) and chest X-ray indicated bilateral opacity with cardiomegaly with lung collapse (Figures 2, 3).

Transthoracic echocardiogram revealed global pericardial effusion with the evidence of RV collapse and clinical signs of cardiac tamponade. Blood, sputum, urine and stool culture did not reveal any growth of any identified pathogen. Serology for melioidosis was also negative.

The patient responded well to emergency pericardiocentesis with subsidence of dyspnoea. He was started with three akurit-4 tablets once per day with 10 mg od of t pyridoxine. The patient was also given tapering dose of steroid regime, oral prednisolone of 60 mg once per day (week 1-4), oral prednisolone of 30 mg once per day (week 5-8), oral prednisolone of 15 mg once per day (week 9-10), and oral prednisolone of 5 mg od for one week. The patient was discharged with no recurrence of symptoms or any signs of deterioration during follow-up visits, eight weeks after the start of therapy.

**Figure 4:**
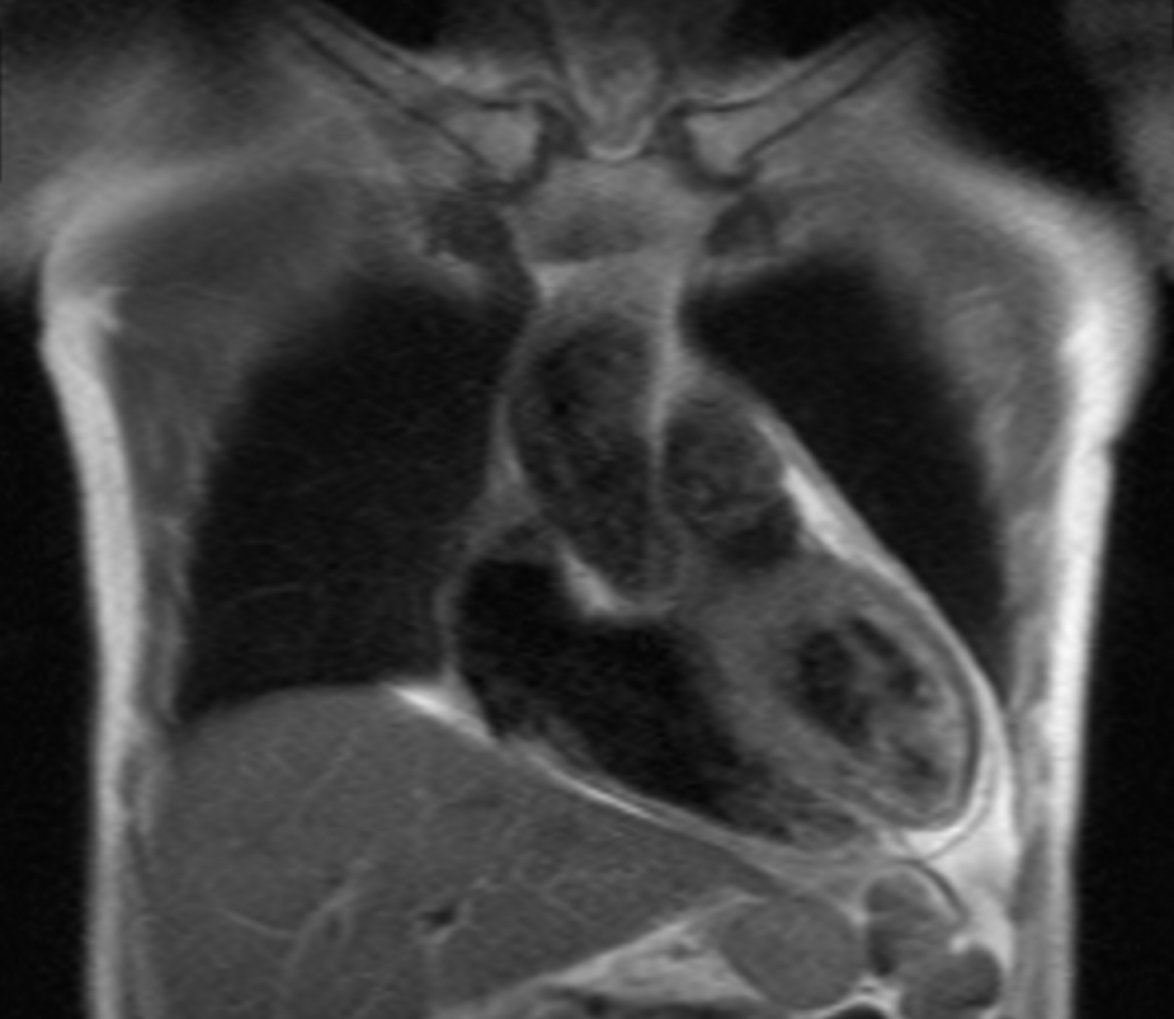
MRI of cardiac showing thickening of pericardium suggestive of constrictive pericarditis.

Repeated echocardiography was planned after the completion of antibiotic. Subsequent serial echocardiography finding showed thickening of pericardium with resolution of pericardial effusion. MRI of cardiac was done showing evidence features of constrictive pericarditis (Figure 4). No hospitalization for similar problem was documented following discharge. We continued to follow up with this patient at our cardiac and respiratory clinic and the patient remained well after discharge.

Based on the exudative effusion and echocardiographic finding, the patient was diagnosed with constrictive pericarditis secondary to Mycobacterium tuberculosis. Written informed consent was obtained from the patient for the publication of this case report.

## Discussion

Pericardial tuberculosis (TB) is a rare presentation of extrapulmonary TB. Extrapulmonary tuberculosis usually develops in 20% of patients with TB infection. It has been estimated that around 1-4% incidence of TB pericarditis commonly occurs via dissemination of lung, spine, sternum, mediastinal lymph node, as well as during milliary infection [6].

Tuberculosis pericarditis is a lethal condition and can be life threatening if prompt treatment is delayed. The mortality rate ranges from 14 to 40% and pyopericardium or purulent pericarditis is rare. The clinical manifestation of tuberculous pericarditis can be non-specific and varies with symptoms of fever, weight loss, night sweats and fatigue. However, it is commonly presented with cough, reduced effort tolerance and chest pain [7]. In some cases, it can be presented with chronic cardiac compression mimicking heart failure or may be presented acutely with cardiac tamponade [8]. This patient was admitted with symptoms mimicking chronic heart failure where he complained of worsening effort tolerance for a one-month duration and was subsequently diagnosed as cardiac tamponade that requires emergency pericardiocentesis. Such complication can be averted by early diagnosis and prompt treatment in tuberculous pericardial empyema which can be life-saving. However, it is often difficult. Active pulmonary TB and pleural effusion maybe observed in 30% of cases with tuberculous pericarditis while 90% of the cases demonstrated features of active pulmonary TB [7]. Non-specific ST wave abnormalities were found in almost all cases with tuberculous pericarditis and other changes include small ECG voltage and electrical alternans [9].

Progression of tuberculous pericarditis can be further divided into four stages. It has been described as: Stage 1, dry stage with early immune response and exudation of fibrinous material; Stage 2, effusive stage of serosanguineous fluid; Stage 3, absorptive stage with pericardial thickening and granulomatous caseation, and lastly Stage 4, constrictive stage caused by scarring effect. The case report of this study was observed and found to be consistent with the previous reported cases where most of the cases yielded straw-coloured fluid and the patient in this study was in the effusive stage during early presentation where the fluid drain was found to be serosanguinous as expected in tuberculous pericarditis. However, we also noticed the presence of purulent pericardial effusion that might suggest TB pyopericardium [7,10].

As the mortality of purulent pericarditis is high, it is important to have early recognition and diagnostic investigation. Aggressive intervention, which includes urgent pericardiotomy and early initiation of tuberculosis treatment, is associated with higher rates of successful recovery. It will result in the formation of fibrin, septations and granulomas with adherence and thickening of leaflets, evolving to chronic constrictive pericarditis if the effusion was not effectively drained from pericardial space [11]. This is based on the latest recommendation of extra-pulmonary tuberculosis treatment. This patient was started with a six-month course of anti-TB medication, with a combination of isoniazid, rifampicin, ethambutol and pyrazinamide for two months, subsequently isoniazid and rifampicin for four months. This regime of treatment has been shown to be highly effective based on a few previous trials [12].

Besides oral anti-tuberculosis, this patient was also started with oral prednisolone as part of the treatment for tuberculous pericarditis. The addition of steroid in the treatment of tuberculous pericarditis has remained controversial and inconclusive. A few reviews on the use of corticosteroids have shown that it reduced the mortality rate and reaccumulated of fluid after 18-24 months of follow-up, however, the drawback of the reviews is that, the small sample size renders the results to be inconclusive [13]. Multicentre randomized trials are needed to address the question. If there is re-accumulation of pericardial effusion, pericardial window is one way to relieve as well as to prevent the development of cardiac tamponade. Pericardiectomy is the treatment of choice to treat the constrictive pericarditis and proper follow-up with serial echocardiography is beneficial to monitor the progression of the disease.

Learning points/take home messages

• Extra-pulmonary TB infection involving purulent pericarditis is a rare manifestation of the disease.

• High degree of suspicion is crucial especially in areas with high endemic state although the presentation is not typical.

• Complete and permanent drainage of the effusion would be the best way to avoid re-accumulation of pericardial fluid.

• The key principles of the management are early diagnosis, and control the infection with appropriate and adequate anti-tuberculosis drug as well as corticosteroid.

## Conclusions

In conclusion, we herein describe an unusual case of purulent TB pericarditis. As we know, TB pyopericardium is rarely presented with TB infection. It also reflects how diagnosis can be delayed if there is no initial suspicions and lack of important history of gastrointestinal symptoms that could aid the diagnosis. Co-morbid conditions play an important role especially in this patient who had a poor diabetic control background. This case report adds further information to the current body of knowledge in disseminated TB infection with the presence of purulent pericarditis.
